# Typical Imaging Features of Perianal Mucinous Adenocarcinoma

**DOI:** 10.5334/jbsr.4095

**Published:** 2026-02-19

**Authors:** Hyeji Kim, Seung Soo Kim, Dong Hyun Kang

**Affiliations:** 1 Department of Radiology, Soonchunhyang University College of Medicine, Cheonan Hospital, Cheonan-si, Republic of Korea; 2 Department of Colorectal Surgery, College of Medicine, Soonchunhyang University Cheonan Hospital, Cheonan-si, Republic of Korea

**Keywords:** mucinous adenocarcinoma, perianal region, computed tomography, magnetic resonance imaging, positron emission tomography computed tomography

## Abstract

*Teaching point:* Typical imaging features of perianal mucinous adenocarcinoma are multilocular cystic mass around the anus, with peripheral irregular enhancement, calcification, and slightly higher signal intensity than muscle on T1-weighted imaging.

## Case History

**Figure 1:**
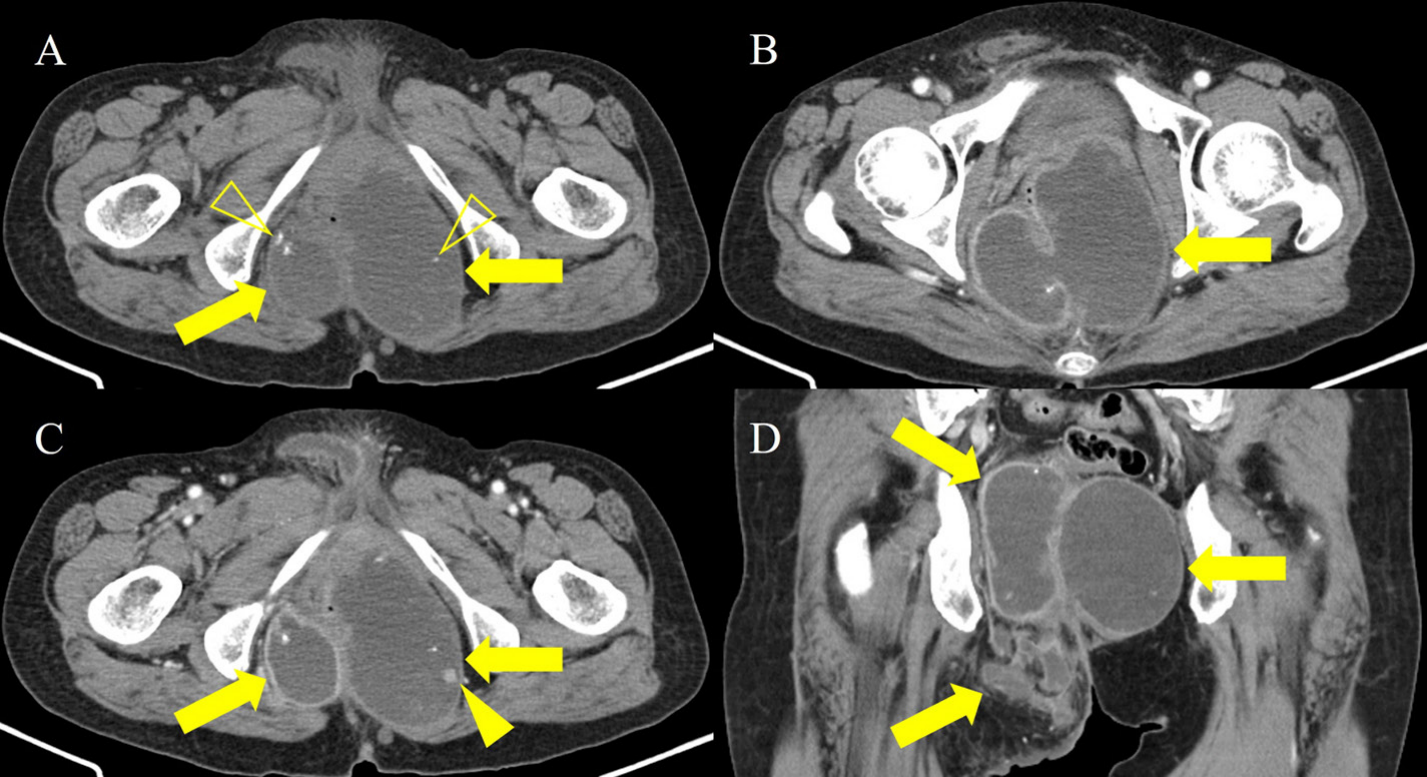
CT images demonstrating multilocular cystic mass around the rectum and anus.

**Figure 2:**
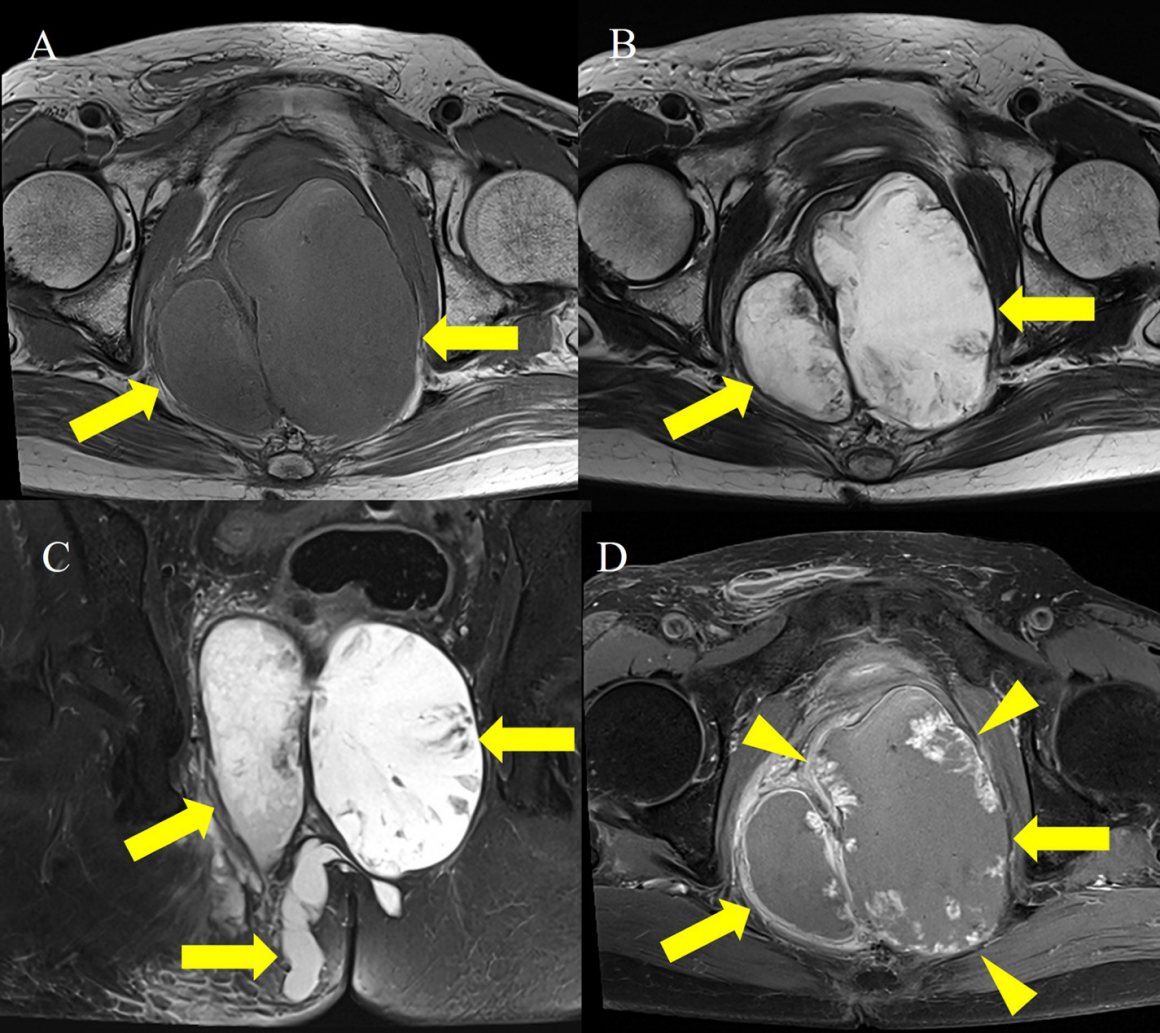
MRI images showing multilocular cystic mass with T1 hyperintensity and peripheral irregular enhancement.

**Figure 3:**
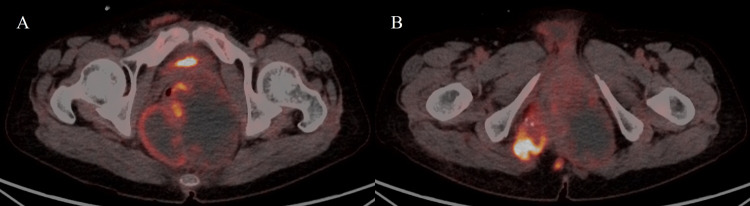
Fluorodeoxyglucose uptake in the cystic mass.

A 65-year-old woman was referred to the hospital for evaluation of a painful perianal mass. Her past medical history was unremarkable. The serum white blood cell count (10,610/mm^3^) and C-reactive protein level (77.08 mg/L) were slightly elevated, but tumor markers were within the normal range, including carbohydrate antigen (CA 19-9), 27.7 U/ml, and carcinoembryonic antigen (CEA), 4.85 ng/ml. Contrast-enhanced computed tomography (CT) was performed for evaluation of the perianal mass. An axial and coronal reformatted contrast-enhanced CT image (Figure 1) demonstrated a 13-cm multilocular cystic mass (arrows) around the rectum and anus, with calcifications (open arrowheads) and an enhancing solid portion (arrowhead). On magnetic resonance imaging (MRI), the multilocular mass (arrows) showed slightly high signal intensity (SI) compared to muscle on axial T1-weighted imaging (T1WI) (Figure 2A) and marked high SI on axial T2-weighted imaging (T2WI) and coronal fat-suppressed T2WI (Figure 2B and 2C). Axial gadolinium-enhanced fat-suppressed T1WI (Figure 2D) revealed peripheral irregular enhancement (arrowheads) of the mass. Positron emission tomography-CT (PET-CT) (Figure 3) showed fluorodeoxyglucose uptake (maxSUV, 11.3) in the mass periphery. The patient underwent mass excision, and the final diagnosis was perianal mucinous adenocarcinoma (PMAC).

## Comment

PMAC is an extremely rare malignant neoplasm that represents approximately 2–19% of all anal carcinomas, which represent 2% of all neoplasms of the gastrointestinal tract [1]. Although the exact histogenesis of PMAC is unclear, the tumor may arise from an anal gland or a chronic fistulous tract. The average age of patients with this condition is 55 years, and it is slightly more common in men [1].

Mucinous adenocarcinoma contains abundant extra-cellular mucin and demonstrates typical CT and MRI features. PMAC typically shows multiple conglomerated cystic masses around the anus, with calcifications on CT. Locules filled with extracellular mucin show higher SI than muscle on T1WI, and SI can differ among them. PMAC usually reveals marked high SI on T2WI. PET-CT may have limitations in evaluating mucinous adenocarcinoma, but fluorodeoxyglucose uptake can be observed [1]. Differential diagnosis from perianal abscess, tail gut cyst, sacrococcygeal teratoma, and pseudomyxoma involving the pelvic retroperitoneum can be difficult. However, a high SI with various levels among locules on T1WI and peripheral irregular enhancement on contrast-enhanced imaging can be helpful in differentiating PMAC from other tumors [1]. Patients with PMAC larger than 5 cm have a poor prognosis [1].
